# Tale of two zones: investigating the clinical outcomes and research gaps in peripheral and transition zone prostate cancer through a systematic review and meta-analysis

**DOI:** 10.1136/bmjonc-2023-000193

**Published:** 2024-04-03

**Authors:** Amin Ali, Thiraviyam Elumalai, BhanuPrasad Venkatesulu, Lauren Hekman, Hitesh Mistry, Ashwin Sachdeva, Pedro Oliveira, Noel Clarke, Esther Baena, Ananya Choudhury, Robert G Bristow

**Affiliations:** 1 Oncology Department, Lancashire Teaching Hospitals NHS Foundation Trust, Preston, UK; 2 Department of Clinical Oncology, The Christie NHS Foundation Trust, Manchester, UK; 3 Prostate Oncobiology, Cancer Research UK Manchester Centre, The University of Manchester, Manchester, UK; 4 Translational Oncogenomics, Cancer Research UK Manchester Centre, The University of Manchester, Manchester, UK; 5 Oncology Department, Addenbrooke's Hospital, Cambridge, UK; 6 Radiation Oncology Department, Loyola University Chicago, Chicago, Illinois, USA; 7 Department of Urology, Loyola University Chicago, Chicago, Illinois, USA; 8 School of Health Sciences, The University of Manchester, Manchester, UK; 9 Department of Surgery, The Christie Hospital NHS Trust, Manchester, UK; 10 Division of Cancer Sciences, The University of Manchester, Manchester, UK; 11 Department of Urology, Salford Royal NHS Foundation Trust, Salford, UK; 12 Department of Pathology, The Christie Hospital NHS Trust, Manchester, UK

**Keywords:** Prostate cancer

## Abstract

**Objective:**

To assess pathological characteristics, clinical features and outcomes of patients diagnosed with peripheral zone (PZ) and transition zone (TZ) prostate cancer after prostatectomy.

**Methods and analysis:**

We systematically reviewed PubMed, EMBASE and MEDLINE. Primary endpoints were biochemical relapse-free survival (bRFS) and distant metastases rate; secondary endpoints included clinical and pathological features.

**Results:**

Ten retrospective cohort studies were identified, six reported HRs for bRFS between PZ and TZ tumours. Patients with TZ tumours had significantly better bRFS (pooled HR 0.57 (0.47, 0.68)) than those with PZ tumours. Two studies reported a lower proportion of distant metastasis in patients diagnosed with TZ tumours compared with PZ tumours (1.5% vs 4.9% (median follow-up 7.0 years) and 0% vs 5% (median follow-up 7.8 years)). PZ tumours presented higher Gleason group and T staging more frequently, while TZ tumours were associated with higher prostate specific antigen levels at diagnosis.

**Conclusion:**

PZ tumours were associated with poorer prognostic clinical features and outcomes. Despite adjusting for poor prognostic clinical features, PZ tumours consistently showed worse clinical outcomes than TZ tumours. Our systematic review underscores the need for further research comparing PZ and TZ prostate cancer to understand the underlying differences and refine clinical practice.

**WHAT IS ALREADY KNOWN ON THIS TOPIC**

- The existing body of scientific knowledge on the topic of peripheral zone (PZ) and transition zone (TZ) prostate cancer highlighted variations in clinical outcomes but lacked a comprehensive understanding of the underlying differences and their implications. Previous studies had indicated differences in biochemical relapse-free survival (bRFS) and clinical features between PZ and TZ tumours, raising the need for a systematic investigation.

**WHAT THIS STUDY ADDS**

- Our systematic review and meta-analysis contribute significantly to the understanding of PZ and TZ prostate cancer in the context of patients treated with surgery majority in the pre-MRI era. We have demonstrated that patients with TZ tumours exhibit notably better bRFS and subsequent distant metastases-free outcomes than those with PZ tumours. Furthermore, our study revealed that PZ tumours are associated with higher Gleason group and T staging, while TZ tumours are linked to higher prostate specific antigen levels at diagnosis. These findings provide a comprehensive insight into the distinct characteristics and clinical outcomes associated with PZ and TZ prostate cancer.

**How this study might affect research, practice, or policy**

- The implications of our study should be considered with caution. Clinically, our findings suggest the potential importance of considering the specific zone of origin when assessing prostate cancer prognosis and planning treatment strategies, especially for patients treated with surgery. While this study provides insights specific to this historical context, it does not directly inform contemporary treatment decisions. Therefore, any influence on the development of tailored therapeutic approaches for PZ and TZ tumours should be carefully considered within the limitations of our study. Moreover, it highlights the need for further research to delve into the underlying differences and refine clinical practice, potentially leading to more personalised and effective management of prostate cancer.

## Introduction

The treatment of localised prostate cancer (PCa) is based on risk assessment using clinicopathological factors, including clinical stage, prostate specific antigen (PSA) level and histological features. PCa is, however, genetically and clinically heterogeneous. Molecular profiling of PCa increases the precision in determining prognosis and individualised treatment.^1–3^ Some international guidelines have adopted the use of tissue-based molecular characteristics.^4 5^ Other pathological PCa features associated with poor prognosis include extracapsular extension, seminal vesicle invasion and multifocality.^6–9^

In addition, the prostatic zone of tumour origin is also prognostic. The prostate is composed of four main areas: anterior fibrous septal area, transition zone (TZ), peripheral zone (PZ) and central zone (CZ); the latter three contain glandular tissue with more than 95% of tumours located in PZ or TZ.^10 11^ Rare CZ tumours are aggressive and are likely to spread via the ejaculatory duct and seminal vesicles.^12^ Compared with TZ tumours, PZ tumours are associated with adverse pathological features (eg, higher Gleason score, extracapsular extension and seminal vesicle invasion in PZ than TZ) and worse prognosis.^13–18^ However, Teloken *et al* 15 have shown that high-grade TZ tumours are independently prognostic with improved biochemical relapse-free survival (bRFS) when compared with corresponding high-grade PZ tumours, suggesting that there are biological differences in zonal tumour location.

For the past few decades, there has been a better understanding of the molecular differences between the normal and tumour prostate zones.^19^ Transcriptomic studies have demonstrated differences in gene expression between PZ and TZ tissue. Sinnott *et al* 20 demonstrated gene signatures that differ between PZ and TZ tissue in malignant and non-malignant samples. The integration of transcriptomics and metabolomics analysis revealed that PZ tissue is associated with altered lipid metabolism in keeping with lipo-rich priming in carcinogenesis. Despite such clinical and molecular differences and their associated prognostic potential, management decisions remain agnostic to the tumour zone of origin.

In this systematic review and meta-analysis, using data from 18 radical prostatectomy retrospective studies, we evaluated the pathological features and prognostic value of the primary tumour location based on the zone of origin in localised PCa. We then consolidated this data from 10 studies to provide an updated and comprehensive analysis of the impact of primary tumour location on PCa outcomes.

## Methodology

### Evidence acquisition

#### Search strategy

**Figure 1:**
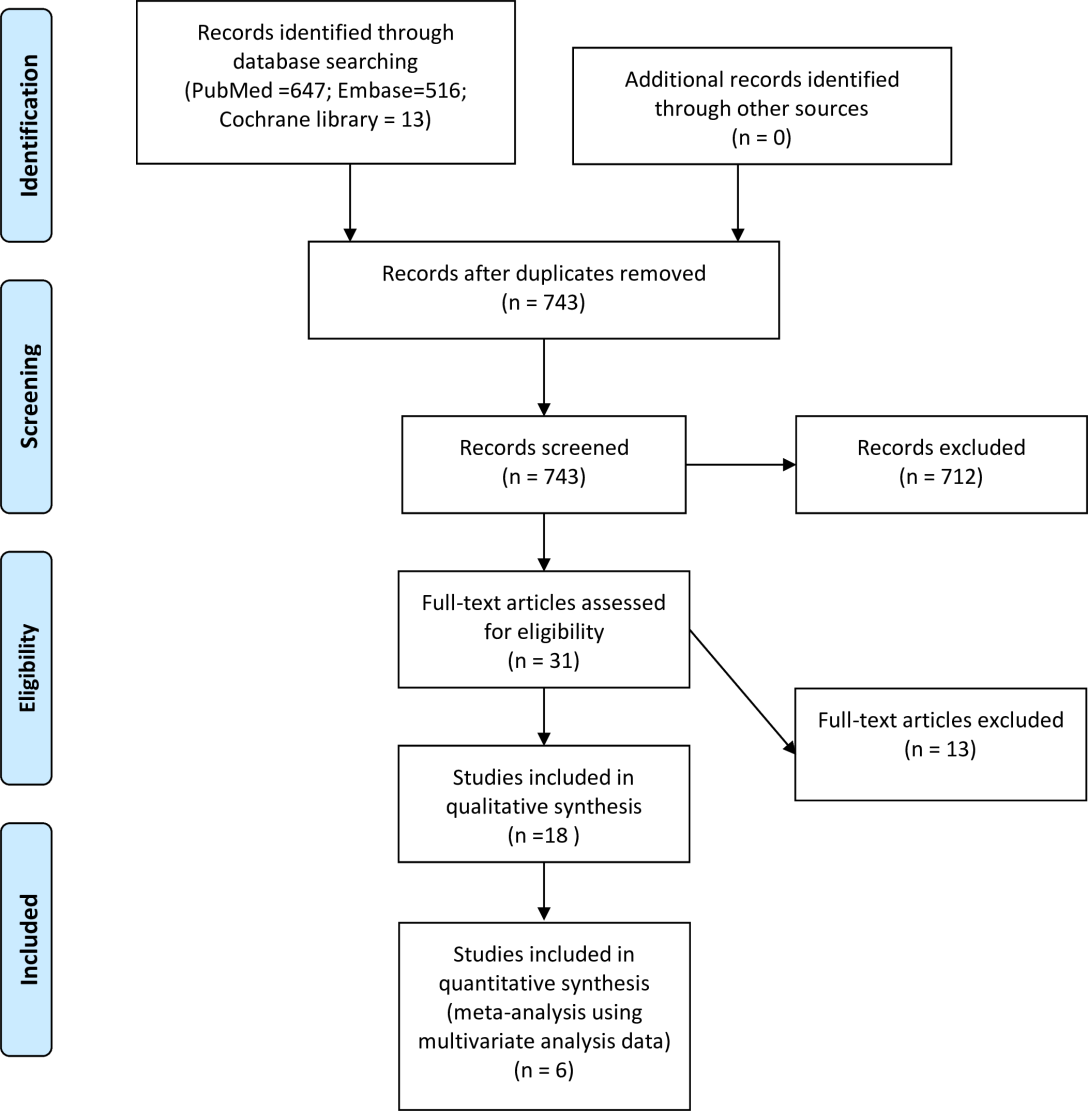
Overview of trials search and selection.

We conducted a systematic review following the Preferred Reporting Items for Systematic Reviews and Meta-analyses (PRISMA) guidelines.^21^ Our search spanned the following databases PubMed, Cochrane Central and EMBASE with the following search terms: PZ, TZ and PCa (figure 1). The search strategy with the MESH terms is provided in online supplemental file 1. The duration of the search spanned from the beginning of the databases up to 21 July 2023. This search did not involve an assessment of conference abstracts and unpublished literature. BPV and TE undertook the database search independently, and any disagreements were resolved through mutual discussion.

10.1136/bmjonc-2023-000193.supp1 Supplementary data

#### Study selection

Studies were deemed eligible if these included men with localised PCa within any prospective trial, retrospective study or cohort study reporting outcomes between PZ and TZ tumours. Only English-language articles were assessed for eligibility. Any duplicates were removed, and titles were screened for inclusion. Potentially relevant reports were subjected to a full-text review, and the relevance of the reports was confirmed after the data extraction process.

#### Data extraction

Data on studies, patients, clinical, pathological characteristics and clinical outcomes were independently extracted by two authors (BPV and TE). We extracted the following variables from the included studies: first author’s name, publication year, country of research, study design, number of patients included, age, tumour characteristics (T stage, baseline PSA level and Gleason grade group), duration of follow-up, biochemical recurrence free survival, metastasis-free survival and overall survival. The proportion of patients with PCa according to zone and countries were calculated based on the total numbers from each study. When there is more than one study from the same institution, the latest and largest cohort of patients was selected for analysis.

#### Statistical analysis

The descriptive analysis was reported as percentages, and continuous variables were reported as medians with an IQR. The HRs were represented with corresponding forest plots comparing differences in outcomes between the PZ and TZ. We included only studies that provided an HR for biochemical recurrence adjusted for confounding variables. Pooled effect estimates were presented by fixed effect and random effect meta-analysis models. Study heterogeneity was assessed using the inconsistency index (I^2^-statistic) with values of 0%–30%, 31%–60%, 61%–75% and 76%–100% indicating low, moderate, substantial and considerable heterogeneity, respectively. A funnel plot was utilised to assess potential publication bias.

## Results/evidence synthesis

Figure 1 (PRISMA flowchart) presents the article selection process. PubMed and MEDLINE were used to identify relevant studies. We initially identified 1,176 potential studies from the three databases (647 were from PubMed, 516 were from MEDLINE and 13 were from Cochrane library). After that, 16 cohorts and 2 case–control studies involving 18,067 participants were included. A meta-analysis was performed on six studies with sufficient data.

### Summary of included studies

The patient characteristics of the 18 included studies are presented in table 1. The years of publication ranged from 1991 to 2020, with six conducted in the USA, four in Germany and three each in Australia and Japan, respectively. Eleven studies reported follow-up data. The length of follow-up ranged from 3 to 20 years.

**Table 1:** Impact of spatial tumour distribution (transition zone vs peripheral zone tumours) on histological features, PSA trend and clinical outcome in radical prostatectomy PCa cohorts. All comparison is transition zone vs peripheral zone tumours unless stated

Publication	Patient cohort (n)	Proportions (%)	T stage (%)	Gleason score	Mean/median PSA (ng/mL)	Clinical outcome (%)
*Lee *et al*, 1991^46^	Cross-sectional (116)	7 vs 93	cT2: 76 vs 81 ≥cT3: 20 vs 19	Mean: 6.2 vs 7.4	N/A	N/A
Stamey *et al*, 1998^47^	Cross-sectional (791)	14 vs 86	cT1c=25 vs 75* cT2=9 vs 92	NA	NA	NA
Noguchi *et al*, 2000^23^	Case–control (158)	50 vs 50 (matched)	cT1c=73 vs 2 cT2=27 vs 78	Gleason grade ≥4/5=35 vs 30%	18.2 vs 21	5 years bRFS: 71.5 vs 49.2
Shannon *et al*, 2003^48^	Case–control (152)	50 vs 50 (matched)	N/A	Gleason grade 4/5≥50%: 54% vs 91%	N/A	N/A
Augustin *et al*, 2003^49^	Cross-sectional (186)	25 vs 75	≤pT2: 74 vs 56 ≥pT3: 26 vs 44	Gleason ≤6: 78 vs 37% Gleason ≥7: 22 vs 63%	15.5 vs 10.9	N/A
Augustin *et al*, 2003^29^	Cross-sectional (307)	21 vs 79	≤pT2: 76 vs 55 ≥pT3: 24 vs 45	Gleason ≤6: 73 vs 37% Gleason ≥7: 27 vs 63%	14.8 vs 11	5 years bRFS: 80 vs 70
Steuber *et al*, 2005^28^	Cross-sectional (1990)	11 vs 89	≤pT2: 73 vs 64 ≥pT3: 27 vs 36	Gleason 6: 78 vs 66% Gleson 3+4: 16 vs 23% Gleason ≥4+3: 7 vs 10%	14.0 vs 9.3	N/A
Steuber *et al*, 2006^27^	Cross-sectional (945)	12 vs 88	≤pT2b: 82 vs 69 ≥pT3a: 27 vs 36	Gleason 6: 74 vs 65% Gleson 3+4: 20 vs 24% Gleason ≥4+3: 5 vs 11%	14 vs 8	N/A
Sakai *et al*, 2006^26^	Cross-sectional (134)	20 vs 80	≤pT2: 63 vs 48 ≥pT3: 37 vs 52	Mean Gleason sum: 6.2 vs 5.9	16.1 vs 12.8	N/A
Chun *et al*, 2007^25^	Cross-sectional (1262)	9 vs 91	NA	Gleason ≤6: 57 vs 39% Gleason ≥7: 43 vs 61%	17.7 vs 10.5	N/A
†Cohen *et al*, 2008^12^	Cross-sectional (726)	7 vs 90 vs 3	N/A	Gleason ≤6: 45 vs 18 vs 0% Gleason 7: 49 vs 78 vs 73% Gleason ≥8: 6 vs 3 vs 27%	7.8 vs 10.8 vs 11.8	2 years bRFS: 72.7 vs 80.9 vs 38.3
King *et al*, 2009^17^	Cross-sectional (494)	18 vs 82	cT1c: 78 vs 69% cT2: 22 vs 49%	Gleason 6: 24 vs 18% Gleason 3+4: 46 vs 63% Gleason ≥4+3: 30 vs 21%	10.8 vs 7.4	5 years bRFS: 85 vs 77
Iremashvili *et al*, 2012^13^	Cross-sectional (1188)	11 vs 89	cT1c: 71 vs 68% cT2: 29 vs 32%	Gleason 6: 54 vs 41% Gleason 3+4: 28 vs 35% Gleason ≥4+3: 18 vs 24%	6.6 vs 5.6	5 years bRFS: 91 vs 82%
Lee *et al*, 2015^14^	Cross-sectional (1354)	17 vs 83	cT1c: 75 vs 49% cT2: 24 vs 51%	Gleason ≤6: 72.2 vs 72.6% Gleason 3+4: 50 vs 54% Gleason ≥4+3: 28 vs 27%	12.1 vs 7.8	5 years bRFS: 80 vs 72 5 years PCM: 99 vs 97
*Teloken *et al*, 2017^15^	Cross-sectional Gleason ≤3+4 (4374)	25 vs 75	N/A	Gleason ≤6: 41 vs 19% Gleason 3+4: 59 vs 81%	7.5 vs 6.3	5 years bRFS: 94 vs 95
	Gleason ≥4+3 (2677)	ten vs 90	N/A	Gleason 4+3: 75.1 vs 73.5% Gleason ≥4+4: 25 vs 26.5%	11 vs 8.7	5 years bRFS: 86 vs 76
Asvadi *et al*, 2018^24^	Cross-sectional (323)	23 vs 77	≤pT2: 64 vs 57% ≥pT3: 36 vs 42%	Gleason 3+3: 12 vs 8% Gleason 3+4: 59 vs 51% Gleason ≥4+3: 19 vs 30%	7.7 vs 5.9	N/A
Takamatsu *et al*, 2019^18^	Cross-sectional (638)	46 vs 54	≤pT2: 75 vs 62% ≥pT3: 25% vs 38%	Gleason 3+3: 14 vs 9% Gleason 3+4: 37 vs 32% Gleason ≥4+3: 49 vs 59%	>10: 25% vs 15%	7 years bRFS: 88 vs 80
Sato *et al*, 2020^16^	Cross-sectional (252)	37 vs 63	≤pT2: 69 vs 48% ≥pT3: 31 vs 52%	Gleason 3+3: 24 vs 9% Gleason 3+4: 53 vs 45% Gleason ≥4+3: 23 vs 46%	7.7 vs 8.3	8 years bRFS: 82 vs 53

*Comparison between TZ vs PZ/CZ.

†Comparison between TZ vs PZ vs CZ.

bRFS, biochemical relapse-free survival; CZ, central zone; N/A, not available; PCa, prostate cancer; PCM, prostate cancer mortality; PSA, prostate specific antigen; PZ, peripheral zone; TZ, transition zone.

### Clinical and pathological features in prostate zones

A total of 18,067 patients from 16 cross-sectional and 2 case–control studies were available for analysis. The definition and demarcation of PZ and TZ tumours varied among studies, as detailed in online supplemental table 1. Some studies defined straddling tumours as either PZ or TZ based on specific criteria, where the majority of the index lesions were in that zone, with tumour areas ranging from 50% to 80%, while others excluded them. The majority of tumours reported were within the PZ (83%). Fourteen of 18 reported studies with Gleason grade group and nine reported studies with tumour staging showed significantly higher grading and stages respectively, in patients diagnosed with PZ tumours compared with TZ tumours (table 1). However, PSA levels were significantly higher in TZ tumours compared with PZ tumours in 11 out of 15 studies that reported this measurement. There has also been variation in the reporting of other prognostic pathological features such as extracapsular extension, seminal vesicle invasion, post-surgical margin positivity, index lesion size, intraductal component and lymph node involvement (online supplemental table 1). There is a higher proportion of patients who underwent prostatectomy with TZ tumour in Japan (41%) compared with Australia (18%), USA (15%) and Germany (11%) (online supplemental table 2).

### Clinical outcome endpoints

**Figure 2:**
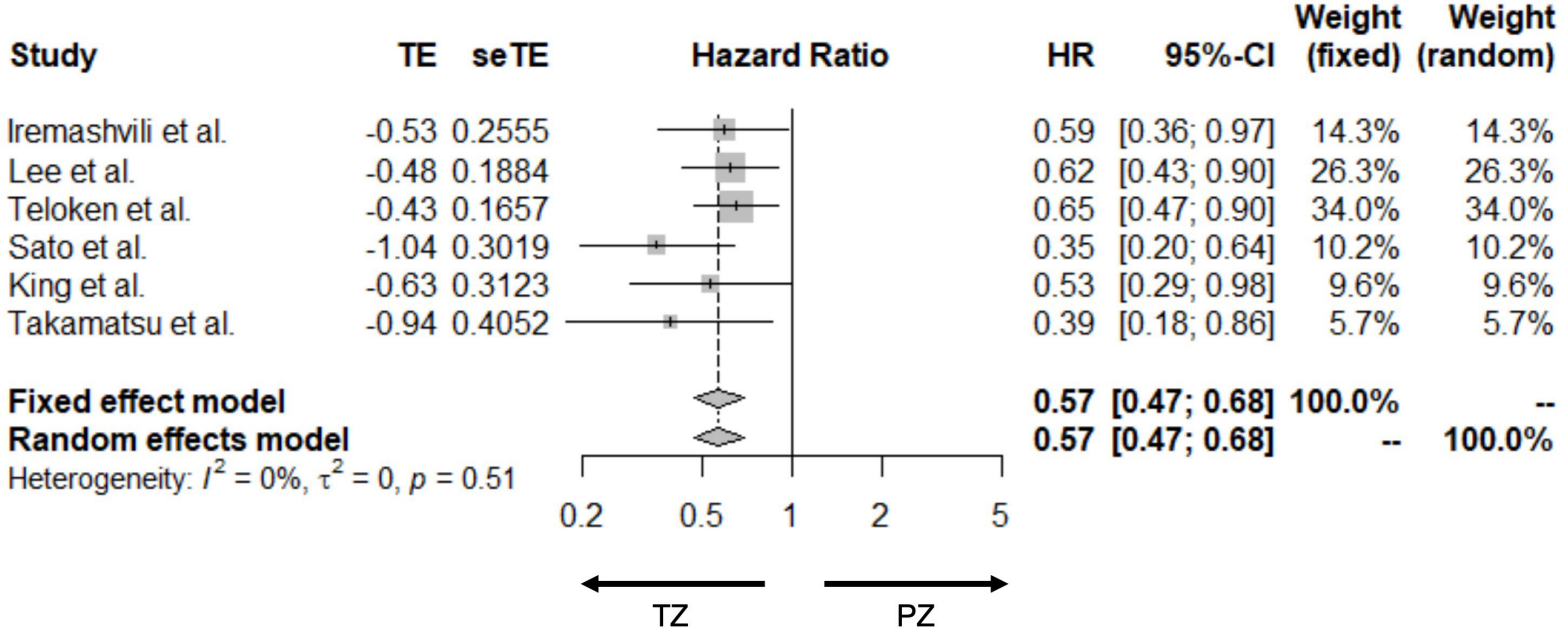
Meta-analysis (Forest Plot) of six studies assessing biochemical recurrence in patients with prostate cancer in the peripheral zone versus transition zone.

**Figure 3:**
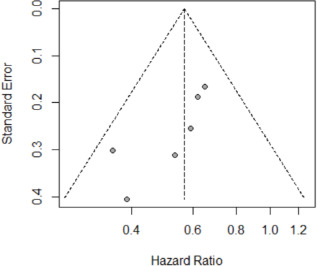
Funnel plot for publication bias of biochemical recurrence meta-analysis.

Ten studies reported bRFS, two reported metastatic-free survival and one overall survival (table 1). Only six cross-sectional studies reported HRs for bRFS and were used to assess the association between prostate zones and bRFS. The HRs from the six cross-sectional studies and pooled HRs are presented in figure 2. Overall, our study suggested a statistically significant relationship between prostate tumour location based on zones and biochemical recurrence risk, the pooled HR (95% CI) was 0.57 (0.47 to 0.68). We observed no evidence of heterogeneity (I^2^=0.0%, p=0.51) (figure 2). All studies in the meta-analysis adjusted for tumour location, Gleason scores, PSA and tumour volume/proportion, while four studies (66.6%) adjusted for extracapsular spread, seminal vesicle and lymph node involvement. Half the studies adjusted for positive margins, age and staging, while individual studies adjusted for bladder neck involvement, lymphovascular invasion, intraductal pathology, body mass index of patients and year of surgery, respectively. The funnel plot showed no strong evidence of publication bias when assessing for publication bias (figure 3). Both studies that reported metastatic-free survival outcomes also showed significantly better prognoses in patients diagnosed with TZ tumours than in PZ tumours. Two studies reported a lower proportion of distant metastasis in patients diagnosed with TZ tumours compared with PZ tumours (1.5% vs 4.9% (median follow-up 7.0 years) and 0% vs 5% (median follow-up 7.8 years)).

## Discussion

Incorporating the seminal work of McNeal,^10^ which laid the foundation for our understanding of prostate zones, this systematic review sheds light on the association between PCa zones of origin and related clinical features and outcomes, based on 16 cohort studies and 2 case–control studies with a total of 18,067 patients. Our meta-analysis of six studies (9,739 patients) suggests that tumours originating in the TZ are associated with better clinical outcomes compared with those in the PZ. Patients with TZ tumours exhibited notably better biochemical recurrence-free survival and a subsequent lower proportion of distant metastases than those with PZ tumours.

These findings suggest that understanding the tumour zone of origin within the prostate could influence treatment decisions and prognosis. Even among high-grade PCa cases, TZ tumours demonstrated better biochemical outcomes, highlighting the importance of considering tumour location when planning treatment strategies. This insight may pave the way for more personalised and targeted approaches to managing PCa. However, there is a notable scarcity of studies that directly address the differences between PZ and TZ PCas. However, several important considerations and limitations need to be explored.

A fundamental issue arising in the context of zonal location is categorising the tumour location into either PZ or TZ. The demarcation between these zones is occasionally ambiguous, and different institutions may adopt varying criteria for categorisation. The demarcation between these zones is often less clear-cut than initially presumed, and our analysis reveals the complexities surrounding this classification. We acknowledge that the categorisation of tumours, especially in the context of anterior tumours can be challenging, Al-Ahmadie *et al* reported a large single-institutional study of 1,312 radical prostatectomies, approximately 15% of tumours were classified as anterior. Notably, nearly half of these anterior tumours were located in the anterior horn of the PZ, highlighting the inadequacy of this simplistic classification in differentiating the zonal origin of PCa. Consequently, only around 5% of tumours were accurately classified as originating in the TZ.^22^ It becomes evident that the simplistic classification of anterior tumours may not effectively differentiate the zonal origin of PCa. Most of the studies included in this meta-analysis mentioned zonal definition criteria for tumours. For example, some studies classified zone of origin based on the largest primary lesion zonal location,^23 24^ the majority of index lesions located in either PZ or TZ^12 13 15 18 25–29^ and the location of the highest Gleason score lesion.^24^ Different cut-offs were also used for the percentage of tumour volume for straddling tumours ranging between more than 50% to 80%, which were classified into respective zones. There was also a study that excluded straddling tumours between two zones^16^ (online supplemental table 1). Our analysis underscores the need for standardised criteria in reporting tumour zonal origin, particularly in the era of precision medicine, to mitigate the inherent variability in defining zonal location and improve the robustness of future research in this field.

The limited evidence we have suggests that tumour location and its surrounding microenvironment may play a role in driving clinical differences, as TZ tumours tend to be associated with better pathological prognostic features. However, only two studies have controlled for known poor pathological prognostic features, which limits the strength of our conclusions.^14 15^ One of the largest studies compared low grade (Gleason grade group ≤2) and high grade (Gleason grade group ≥3) of 4,374 and 2,677 patients, respectively.^15^ Interestingly, TZ tumours were maintained to have a better biochemical outcome in high grade PCa. It is essential to acknowledge that anatomical factors of PZ tumours place them closer to vessels, nerves, the prostatic capsule and seminal vesicles than TZ tumours, which could potentially contribute to a worse prognosis. Besides this, further studies have supported the potential role of tumour location and its surrounding microenvironment in driving these differences in clinical outcomes.^30 31^ Further molecular studies have shown differences between the two zones. The TMPRSS-ERG gene fusion event is the most common in PCa and occurs more frequently in PZ tumours compared with TZ tumours.^32–34^ Gene expression studies have also shown distinct transcriptomic profiles between the zones, for example, non-malignant PZ tissue has higher de novo lipid biosynthesis,^35^ potentially providing a better niche for PCa tumorigenesis. Another recent study also showed higher expression of stromal FOXF2 in non-malignant TZ compared with PZ tissue, facilitating an anti-tumour immunity microenvironment that potentially contributed to the different outcomes between zones.^36^ It is crucial to consider additional future research to investigate the differences in tumour location and account for potential confounding factors to understand better the impact of the tumour’s origin on prognosis and treatment outcomes.

By understanding the tumour zone of origin and the contribution of its microenvironment in tumorigenesis, there is a potential role in treatment stratification in localised PCa.^19^ Besides surgery, radiotherapy is also another curative treatment modality. The utility of MRI has enabled investigating the differences between PZ and TZ tumours after radiotherapy. For example, Song *et al* investigated the MRI changes before and after radiotherapy in 47 PZ tumours and 12 TZ tumours where the apparent diffusion coefficient changes decreased in both zones after treatment.^37^ An earlier study by Amico *et al* reported TZ tumours based on clinical criteria and not MRI scans may benefit from radiotherapy despite having a high PSA level (>20 ng/mL).^38^ With advances in radiological imaging, tumour location could be mapped before treatment. In a more recent study, Asuscion *et al* investigated the use of mpMRI (multiparametric MRI) and 3D magnetic resonance spectroscopy to evaluate the prognostic stratification of localised PCa treated by radiotherapy, including external beam with or without brachytherapy.^39^ The authors reported biochemical recurrence rates at 5 years of 8% for TZ tumours and 18% for PZ tumours. However, confounding factors of other prognostic factors were not taken into consideration. In the era of precision medicine, clinical trials such as FLAME used imaging information for dose escalation radiotherapy to dominant lesions highlighting the reliability and accuracy of mapping tumour location pretreatment.^30^ By integrating information on tumour zonal location, prospective clinical follow-up would provide a better understanding of treatment efficacy based on zones, as it has not been studied before.

A limitation of our review is that most studies were carried out before MRI became part of the routine investigations. In this historical context, the use of transrectal systematic biopsies for diagnosing PCa led to the underdiagnosis of anterior cancers in men with elevated PSA levels. Anterior cancers are now readily detected with MRI and targeted biopsy, which means that they do not occur as often as before and are left undiagnosed to progress over a long period. Additionally, high-grade anterior cancers may have had the opportunity to metastasise before diagnosis, making them ineligible for surgical treatment, in contrast to posterior cancers, which were often diagnosed at an earlier, more treatable stage, highlighting a possible selection bias between PZ and TZ tumours. Therefore, the generalisability of our findings should be approached with caution, particularly when applying them to patients diagnosed after the widespread adoption of pre-biopsy MRI. The introduction of pre-biopsy MRI has likely altered the distribution of anterior and posterior tumours, as demonstrated by Schouten *et al.* 31 Asvadi *et al* have shown the reliability of mpMRI correlating zonal locations of PCa when compared with histological findings from prostatectomy samples.^24^

Another consideration is the time span of the studies included in our review, ranging from early 1990s to 2020. The potential impact of the International Society of Urological Pathology revisions in 2005^40^ and 2014^41^ regarding Gleason grading should not be overlooked. These revisions led to significant grade migration following the update for example, a higher proportion of GGG1 to GGG2.^42 43^ This evolving nature and challenge of PCa grading could impact patient stratification and clinical outcomes when comparing studies conducted over several decades.

Despite the differences in clinical outcomes between patients with TZ tumours having better biochemical recurrence free survival compared with PZ tumours, the cut-off for biochemical recurrence and follow-up visits differs between studies, reflecting the heterogeneity in clinical practice. Also, the intermediate clinical endpoint of clinical recurrence (disease free survival) and metastasis-free survival has shown to be a strong surrogate of overall survival in localised PCa compared with bRFS.^44 45^ Despite that, two studies showed patients with TZ tumours have a significantly lower proportion of distant metastases than patients with PZ. Establishing a standardised definition for tumour location boundaries would facilitate more accurate comparisons between studies and provide a more robust foundation for future research. There has also not been any information reported regarding further treatment in patients with disease recurrence. By following up on the tumour zone of origin, the differential response to treatment in both localised (ie, radical prostatectomy or radiotherapy) and metastatic (ie, androgen receptor signalling inhibitors or chemotherapy) settings may be present.

In conclusion, better clinical outcomes in terms of biochemical recurrence and subsequent distant metastasis were observed in patients with TZ tumours compared with PZ tumours. However, there is a significant lack of research directly addressing the differences between PZ and TZ PCas. Awareness of accurate reporting of tumour zones by pathologists and including such data in clinical trials will improve understanding of the differential impact of biology between tumour zones and the potential benefit of novel therapeutic approaches. Further research is warranted to understand the underlying biology and potentially refine clinical practice. Addressing the scarcity of studies and standardising tumour location definitions will provide a more robust foundation for future research and ultimately contribute to improved patient outcomes.

## Data Availability

No data are available.
