# Supplementary material for: Tale of two zones: investigating the clinical outcomes and research gaps in peripheral and transition zone prostate cancer through a systematic review and meta-analysis
Source: BMJ Oncol. 2024 Apr 3;3(1):e000193. doi: 10.1136/bmjonc-2023-000193 (PMC11234997; doi:10.1136/bmjonc-2023-000193)
Supplement: Supplementary data [file bmjonc-2023-000193supp001.pdf]

Supplementary Table 1. Clinical and pathological characteristics of spatial tumour distribution (transition zone vs peripheral zone tumours) studies

BMI = body mass index; bRFS = biochemical recurrence; DM = distant metastases rate, ECE = extracapsular extension; GG = Gleason Grade; IDC = intraductal carcinoma; LVI = lymphovascular invasion; LNI = lymph node involvement; PSM = post-surgical margin; PSA = prostate specific antigen; SVI = seminal vesicle invasion,

Author	Country	Recruitment Period	Total patients	Zone definition	Clinical features	Pathological features	Clinical outcomes	PSA surveillance	bRFS definition (PSA ng/ml)
[x]Lee et al., 1991(48)	USA	1985-1989	116	Tumour zone classification based on transrectal ultrasound findings. Categorised as outer (PZ and CZ tumours) or inner (TZ tumours) gland. No mention of straddling tumour inclusion/exclusion criteria.	Age	Pathology stage, IDC, ECE, SVI, multifocality, tumour morphology	NA	NA	NA
Stamey et al., 1998(49)	USA	1985-1996	791	Tumour location defined as per index tumour location. No mention of straddling tumour inclusion/exclusion criteria.	Age, PSA, clinical stage	Tumour volume, Gleason percentage	NA	NA	NA
Noguchi et al., 2000(23)	USA	1988-1997	158	Tumour location defined as per index tumour location. No mention of straddling tumour inclusion/exclusion criteria.	Age, PSA, clinical stage	Tumour volume, Gleason percentage, ECE, PSM, LNI, prostate weight	bRFS	Not mentioned	0.07

Shannon et al., 2003(50)	Australia	1998 - 2003	152	Tumour location defined as per index tumour location.<br>No mention of straddling tumour inclusion/exclusion criteria	Age	Tumour volume, Gleason percentage, ECE, PSM, SVI	NA	NA	NA
Augustin et al., 2003(51)	Germany	1994-1997	505	TZ tumours were defined as >50% of index tumour within TZ.<br>Based on Stamey 1998, Noguchi 2000	Age, PSA, clinical stage	Pathology stage, tumour volume, LN, PSM, GG	NA	NA	NA
Augustin et al., 2003(29)	Germany	1994-1997	505	TZ tumours were defined as >50% of index tumour within TZ.<br>PZ tumours were defined as 100% of index tumour within PZ<br>Based on Stamey 1998, Noguchi 2000	Age, PSA, clinical stage	Pathology stage, GG, LN, PSM, prostate weight	bRFS (matched case control)	3/12 post op then annually	0.1
Steuber et al., 2005(28)	Germany	1994-2002	1990	TZ tumours were defined as >70% of index tumour within TZ (McNeal 1988, Erbersdobler 2002).<br>In extensive multifocal TZ tumours, the index tumour was located in TZ or >50% the cancer volume of multiple tumour foci located in the TZ boundary (Augustin 2003).<br>PZ tumours were defined as exclusively within PZ and all the indeterminate origins	Age, PSA, clinical stage	Pathology stage, ECE, SVI, LNI, GG	NA	NA	NA

Steuber et al, 2006(27)	Germany	1997-2003	945	TZ tumours were defined as >50% of index tumour within TZ.<br>PZ tumours were defined as 100% of index tumour within PZ<br>Based on Stamey 1998, Noguchi 2000	Age, PSA, clinical stage	ECE, PSM, GG	NA	NA	NA
Sakai et al., 2006(26)	Japan	1997-2004	172	Tumour locations were defined as >70% of index tumour within either PZ or TZ respectively.<br>Other tumours (indeterminate) were excluded for further analysis.	Age, PSA, clinical stage, positive cores	Pathology stage, GG, SVI, LNI, PSM, LVI, PNI, tumour volume	bRFS	3/12 x 2y then 6/12	0.2
Chun et al., 2007(25)	Germany	1996-2004	1262	TZ tumours were defined as >70% of index tumour within TZ.<br>In extensive multifocal TZ tumours, the index tumour was located in TZ or >50% the cancer volume of multiple tumour foci located in the TZ boundary.<br>PZ tumours were defined as exclusively within PZ and all the indeterminate origins.	PSA	GG, ECE, SVI, LNI	bRFS	3/12 x 1y -> 6/12 x 1y, then annually	0.1
[y]Cohen et al., 2008(12)	Australia	1998-2006	726	Tumour locations were defined as >80% of index tumour within either PZ or TZ respectively.	Age, PSA	Tumour volume, GG, ECE, SVI, ejaculatory duct	bRFS	Annual	0.2

				Other tumours (indeterminate) were excluded for further analysis.		invasion, IDC, LNI, PSM			
King et al., 2009(17)	USA	1989-2000	494	Tumour location defined as per index tumour location.<br>No mention of straddling tumour inclusion/exclusion criteria.<br>Protocol as per Noguchi 2000.	Age, clinical stage, PSA, GG	GG, SVI, PSM, ECE, tumour volume, LNI, prostate weight, year of surgery	bRFS	3/12 x 1y -> 6/12 x 1y, then annually	0.1
Iremashvili et al., 2012(13)	USA	1992-2011	1441	Tumour locations were defined as >70% of index tumour within either PZ or TZ respectively.<br>Other tumours (30-69%) were defined as indeterminate.	Age, PSA	Pathology stage, GG, ECE, PSM, SVI, LNI	bRFS	3/12 x 2y then 6/12	0.2
Lee et al., 2015(14)	USA	1983-2003	1588	Tumour location defined as per index tumour location.<br>No mention of straddling tumour inclusion/exclusion criteria.<br>Protocol as per Noguchi 2000.	Age, clinical stage, PSA, GG	GG, prostate weight, tumour volume, high grade tumour volume, ECE, SVI, LVI, LN	DM, bRFS, PCM (OS)	3/12 x 1y -> 6/12 x 2y, then annually	0.1
[x]Teloken et al., 2017(15)	Australia	1998-2016	7051	Tumour locations were defined as >75% of index tumour within either PZ or TZ respectively.	Age, PSA	Prostate weight, tumour volume, GG, IDC, ECE, PSM, SVI, LN	bRFS	3/12 x 1y -> 6/12 x 1y, then annually	0.2

	USA	2009-2016	323	Tumour locations were define based on radiological mapping (PIRADSv2)<br>No mention of straddling tumour inclusion/exclusion criteria (pathology).	Age, PSA, GG, Radiology (PI-RADSv2, tumour & prostate volume)	Index lesion size, GG, ECE, SVI, pathology stage, prostate weight	NA	NA	NA
Asvadi et al., 2018(24)	Japan	2005-2015	638	Tumour locations were defined as >50% of index tumour within either PZ or TZ respectively.	Age, BMI, PSA, GG, clinical stage	Index lesion size, prostate volume, tumour number, GG, pathology stage, SVI, LN, PSM	bRFS	Not mentioned	0.2
Takamatsu et al., 2019(18)	Japan	2009-2012	270	Tumour location defined as per index tumour location.<br>Tumours straddling between TZ and PZ were excluded	Age, clinical stage, GG, positive cores, NCCN classification	GG, EPE, pathology stage, PSM, IDC, LN, ERG overexpression, PTEN loss, SPINK1 expression	bRFS, DM	Not mentioned	0.2

Supplementary Table 2. PCa proportion by zone location and country.

Country	Author	Zone		Total (n)
		TZ (n)	PZ (n)	
Australia	Cohen(12)	49	655	704
	Teloken(15)	1,099	3,275	4,374
	Teloken(15)	273	2,404	2,677
	Total (Percentage)	1421 (18%)	6334 (82%)	7755 (100%)
Japan	Sakai(26)	24	100	124
	Takamatsu(18)	293	345	638
	Sato(16)	93	159	252
	Total (Percentage)	411 (41%)	603 (59%)	1,014 (100%)
USA	Lee(48)	8	108	116
	Iremashvili(13)	147	1,1141	1,288
	Lee(14)	230	1,124	1,354
	Asvadi(24)	79	244	323
	Total (Percentage)	464 (15%)	2,617 (85%)	3,081 (100%)
Germany	Augustin(29)	63	244	307
	Steuber(28)	222	1,768	1,990
	Chun(25)	115	1,147	1,262
	Total (Percentage)	400 (11%)	3,159 (89%)	3,559 (100%)
**Overall**	**Total (Percentage)**	**2,696 (17%)**	**12,713 (83%)**	**15,409 (100%)**

PUBMED database

1.Query –Peripheral zone

Result: 8,218

("peripheral"[All Fields] OR "peripherally"[All Fields] OR "peripherals"[All Fields] OR "peripheral"[All Fields] OR "peripheric"[All Fields] OR "peripherally"[All Fields]) AND "zone"[All Fields]

2. **Query-Transition zone**

Result: 11,385

("transit"[All Fields] OR "transited"[All Fields] OR "transiting"[All Fields] OR "transition"[All Fields] OR "transitional"[All Fields] OR "transitional"[All Fields] OR "transitioned"[All Fields] OR "transitioning"[All Fields] OR "transitions"[All Fields] OR "transits"[All Fields]) AND "zone"[All Fields]

3. **Query –Prostate cancer OR Prostate neoplasm OR Prostate malignancy OR Prostate tumor**

Result: 210,920

"prostatic neoplasms"[MeSH Terms] OR ("prostatic"[All Fields] AND "neoplasms"[All Fields]) OR "prostatic neoplasms"[All Fields] OR ("prostate"[All Fields] AND "cancer"[All Fields]) OR "prostate cancer"[All Fields] OR ("prostatic neoplasms"[MeSH Terms] OR ("prostatic"[All Fields] AND "neoplasms"[All Fields]) OR "prostatic neoplasms"[All Fields] OR ("prostate"[All Fields] AND "neoplasm"[All Fields]) OR "prostate neoplasm"[All Fields]) OR (("prostat"[All Fields] OR "prostate"[MeSH Terms] OR "prostate"[All Fields] OR "prostates"[All Fields] OR "prostatic"[All Fields] OR "prostatism"[MeSH Terms] OR "prostatism"[All Fields] OR "prostatitis"[MeSH Terms] OR "prostatitis"[All Fields]) AND ("malign"[All Fields] OR "malignance"[All Fields] OR "malignances"[All Fields] OR "malignant"[All Fields] OR "malignants"[All Fields] OR "malignities"[All Fields] OR "malignity"[All Fields] OR "malignization"[All Fields] OR "malignized"[All Fields] OR "maligns"[All Fields] OR "neoplasms"[MeSH Terms] OR "neoplasms"[All Fields] OR "malignancies"[All Fields] OR "malignancy"[All Fields])) OR ("prostatic neoplasms"[MeSH Terms] OR ("prostatic"[All Fields] AND "neoplasms"[All Fields]) OR "prostatic neoplasms"[All Fields] OR ("prostate"[All Fields] AND "tumor"[All Fields]) OR "prostate tumor"[All Fields])

Final query #1 AND #2 AND # 3

Result 722

("peripheral"[All Fields] OR "peripherally"[All Fields] OR "peripherals"[All Fields] OR "periphereal"[All Fields] OR "peripheric"[All Fields] OR "peripherically"[All Fields]) AND "zone"[All Fields] AND

(("transit"[All Fields] OR "transited"[All Fields] OR "transiting"[All Fields] OR "transition"[All Fields] OR "transitional"[All Fields] OR "transitionals"[All Fields] OR "transitioned"[All Fields] OR "transitioning"[All Fields] OR "transitions"[All Fields] OR "transits"[All Fields]) AND "zone"[All Fields]) AND ("prostatic neoplasms"[MeSH Terms] OR ("prostatic"[All Fields] AND "neoplasms"[All Fields]) OR "prostatic neoplasms"[All Fields] OR ("prostate"[All Fields] AND "cancer"[All Fields]) OR "prostate cancer"[All Fields] OR ("prostatic neoplasms"[MeSH Terms] OR ("prostatic"[All Fields] AND "neoplasms"[All Fields]) OR "prostatic neoplasms"[All Fields] OR ("prostate"[All Fields] AND "neoplasm"[All Fields]) OR "prostate neoplasm"[All Fields]) OR (("prostat"[All Fields] OR "prostate"[MeSH Terms] OR "prostate"[All Fields] OR "prostates"[All Fields] OR "prostatic"[All Fields] OR "prostatism"[MeSH Terms] OR "prostatism"[All Fields] OR "prostatitis"[MeSH Terms] OR "prostatitis"[All Fields]) AND ("malign"[All Fields] OR "malignance"[All Fields] OR "malignances"[All Fields] OR "malignant"[All Fields] OR "malignants"[All Fields] OR "malignities"[All Fields] OR "malignity"[All Fields] OR "malignization"[All Fields] OR "malignized"[All Fields] OR "maligns"[All Fields] OR "neoplasms"[MeSH Terms] OR "neoplasms"[All Fields] OR "malignancies"[All Fields] OR "malignancy"[All Fields])) OR ("prostatic neoplasms"[MeSH Terms] OR ("prostatic"[All Fields] AND "neoplasms"[All Fields]) OR "prostatic neoplasms"[All Fields] OR ("prostate"[All Fields] AND "tumor"[All Fields]) OR "prostate tumor"[All Fields]))

EMBASE database

#	Searches	Results
1	Peripheral zone.mp.	3340
2	Transition zone.mp.	5798
3	(Prostate cancer or Prostate neoplasm or Prostate malignancy or Prostate tumor)	153895
4	#1 AND #2 AND #3	322

Cochrane central

Search Name:

Date Run: 21/07/2023 23:40:03

Comment:

ID	Search	Hits
#1	Peripheral zone	441
#2	Transition zone	170
#3	Prostate cancer OR Prostate neoplasm OR Prostate malignancy OR Prostate tumor	18087
#4	#1 AND #2 AND #3	16
